# MRI-guided focused ultrasound (MRgFUS) surgery in conserving therapy of adenomyosis

**DOI:** 10.1186/2050-5736-2-S1-A10

**Published:** 2014-12-10

**Authors:** EN Zhumanova, Yu V Lazutkina, Yu O Gorbenko, AA Ishchenko, EA Chunaeva, AI Ishchenko, EA Mershina, VE Sinitsyn

**Affiliations:** 1 Federal Center of Treatment and Rehabilitation, Russian Federation

## Background

Adenomyosis is a complex disorder that accounts up to one third of gynecological morbidity. Diffuse form of adenomyosis is most difficult to diagnose and treat. All attempts to create a "gold standard" in preserving therapy have not been successful so far. MRgFUS could be a promising technology for organ-preserving treatment of this disease.

Purpose of this study was to determine the efficacy of MRgFUS ablation in organ-preserving treatment of adenomyosis.

## Materials and methods

15 women (25-45 yrs old) with symptomatic adenomyosis were included in the study. Informed consent was obtained in all cases. The diagnosis and the technical ability to follow the procedure protocol were confirmed by MRI. Ablation was performed using ExAblate-2000 (InSightec, Israel) by guidance of 1,5T MRI (General Electric, USA). Control MRI and Doppler mapping were performed immediately after and in 3 months after MRgFUS ablation to evaluate uterine volume and vascularity of myometrium. All the patients completed questionnaires before and 3 months after the procedure.

## Results

12 of 15 pts (80%) reported an improvement of life quality in 3 months. Regression of pain was observed in 80% of cases (12 pts), a decrease of metrorrhagia - in 60% (9 pts). Recurrence of the symptoms in these patients was not observed 6 months after treatment.

In 70% of cases (10 pts) the uterine volume decreased by 33% 3 months after ablation.

In 60% of women (9 pts) non-perfused zones were described using MRI with contrast-enhancement, averaging 26% of the affected areas. Decrease of vascularity was confirmed by Doppler mapping technique.

## Conclusion

MRgFUS ablation may become a method of choice for conserving therapy of the patients with a confirmed diagnosis of adenomyosis especially in reproductive age. This method is non-invasive, does not require hospitalization. This requires further observation and confirmation in a bigger group of patients and in a long-term period.

